# Molecular Characterization and Pathogenicity of a Novel Recombinant NADC30‐Like PRRSV Strain Isolated From Piglets Inducing Intestinal Inflammation, and Construction of Its Infectious Clone

**DOI:** 10.1155/tbed/4893251

**Published:** 2025-12-26

**Authors:** Yafang Lin, Yan Ouyang, Jiayang Zheng, Changguang Xiao, Yang Yang, Qianming Zhao, Yan Zhang, Zongjie Li, Ke Liu, Beibei Li, Donghua Shao, Yafeng Qiu, Zhiyong Ma, Jianchao Wei

**Affiliations:** ^1^ Key Laboratory of Animal Microbiology of China’s Ministry of Agriculture, College of Veterinary Medicine, Nanjing Agricultural University, Nanjing, 210095, China, njau.edu.cn; ^2^ Shanghai Veterinary Research Institute, Chinese Academy of Agricultural Sciences, Shanghai, 200241, China, caas.cn; ^3^ College of Agriculture, Hubei Three Gorges Polytechnic, Yichang, 443000, China

**Keywords:** infectious clone, intestinal tropism, NADC30-like PRRSV, pathogenicity, recombination analysis, viral isolation

## Abstract

Porcine reproductive and respiratory syndrome virus (PRRSV) causes major economic losses in swine production worldwide, characterized by reproductive failure and respiratory disease. In China, the cocirculation of diverse strains drives ongoing viral evolution and the emergence of novel recombinants, posing serious challenges for disease control. Here, we report the isolation and characterization of a novel PRRSV strain (JS‐YZ/2023) from a PRRS‐suspected pig farm. Genomic analysis revealed a unique recombinant pattern between NADC30‐like and HP‐PRRSV lineages, distinct from previously reported recombinants. Experimental infection in piglets demonstrated significant pathogenicity, with persistent fever, severe anorexia, progressive weight loss, and pathological lesions including interstitial pneumonia with lymphocyte infiltration and marked disruption of intestinal villi. Notably, JS‐YZ/2023 exhibited strong intestinal tropism, inducing a local cytokine storm in the small intestine and showing PRRSV‐N protein antigen accumulation in crypt epithelial cells, as detected by immunohistochemistry. In addition, we successfully constructed a full‐length infectious cDNA clone of JS‐YZ/2023 incorporating a green fluorescent protein (GFP) reporter gene (rGFP‐JS‐YZ/2023). This engineered virus provides a valuable tool for investigating the pathogenic mechanisms of NADC30‐like recombinants and facilitating vaccine development.

## 1. Introduction

Porcine reproductive and respiratory syndrome (PRRS), caused by the PRRS virus (PRRSV), is a globally significant disease in *Sus scrofa* responsible for major economic losses in pork production because of its high transmissibility and severe clinical impact [1–3]. PRRSV primarily targets porcine alveolar macrophages (PAMs), leading to reproductive failure—such as abortions and stillbirths in sows—as well as respiratory disease, including pneumonia, in pigs. These effects contribute to decreased herd performance, reduced feed efficiency, and increased mortality [4].

PRRSV is an enveloped, single‐stranded, positive‐sense RNA virus belonging to the family *Arteriviridae*. Its genome is approximately 15 kilobases in length [5], with a 5′ cap and a 3′ polyadenylated tail [6], and it encodes at least 11 open reading frames (ORF) [7, 8]. Virions are spherical, measuring 50–65 nm in diameter, and contain envelope glycoproteins including GP2, GP3, GP4, and GP5 [9, 10]. Two distinct PRRSV species have been identified: *Betaarterivirus suid* 1 (PRRSV‐1) and *Betaarterivirus suid* 2 (PRRSV‐2) [11–13]. This reclassification highlights their genetic divergence and necessitates distinct diagnostic and control strategies. Genetic analysis further divides PRRSV‐2 into multiple lineages. In China, the prevalent lineages include 1, 3, 5, and 8 [14], with Lineage 1.5 represented by strain IA/2014/NADC34 and Lineage 1.8 by NADC30 [15, 16].

Genetic recombination drives PRRSV genomic diversity in China, facilitating immune evasion and increased virulence. Since 2013, NADC30‐like strains (Lineage 1.8) have become dominant, while HP‐PRRSV (Lineage 8.7) has persisted at a prevalence of 10%–15%. These lineages frequently recombine, accounting for more than 48% of recombination events reported in China [17]. Such recombination generates viruses with enhanced fitness. Continuous monitoring remains essential given the risks of altered pathogenicity and vaccine escape. Meanwhile, advances in reverse genetics systems have provided pivotal tools for dissecting PRRSV pathogenesis and developing novel vaccines. This study reports the isolation of a novel recombinant NADC30‐like PRRSV strain circulating in China, characterizes its in vitro growth kinetics and in vivo pathogenicity, and establishes a full‐length cDNA infectious clone with stable green fluorescent protein (GFP) expression. Together, these efforts deepen our understanding of PRRSV pathogenicity and support the development of more effective strategies for outbreak control worldwide.

## 2. Materials and Methods

### 2.1. Cells, Strains, and Vectors

MARC‐145 and BHK‐21 cells were cultured in Dulbecco’s modified eagle medium (DMEM) (Gibco, Thermo Fisher Scientific, Waltham, MA, USA) supplemented with 10% fetal bovine serum (FBS) (Biological Industries, Sartorius, Kibbutz Beit Haemek, Israel) and antibiotics (penicillin/streptomycin) (Yeasen Biotechnology, Shanghai, China). PAMs, isolated by lung lavage from specific pathogen‐free piglets, were cultured in Roswell Park Memorial Institute 1640 medium (Gibco, Thermo Fisher Scientific), also supplemented with 10% FBS and antibiotics. The low copy number plasmid pACYC177‐CMV was maintained at −80°C in our laboratory.

### 2.2. Virus Isolation

PRRSV‐positive lung samples were collected from a PRRS‐suspected pig farm in Jiangsu Province that had not been inoculated with PRRSV vaccine and had no neighboring pig farms within 3 km. The sows showed reproductive disorders, including abortions, stillbirths, and mummified fetuses, while piglets exhibited clinical symptoms such as fever, coughing, depression, and diarrhea. For virus isolation, lung homogenates from diseased piglets were inoculated onto MARC‐145 cell monolayers. Cells were maintained at 37°C with 5% CO_2_ in DMEM supplemented with 10% FBS and 1% antibiotics. Viral supernatant was harvested immediately upon observation of the characteristic grape‐like cluster cytopathic effect (CPE). In cases where CPE did not appear, supernatant was collected at 4 days postinfection (dpi) and stored at −80°C.

### 2.3. Indirect Immunofluorescence Assay

MARC‐145 cells infected with JS‐YZ/2023 at a multiplicity of infection (MOI) of 0.01 were analyzed by indirect immunofluorescence assay at 24 h post‐infection (hpi). Cells were fixed with 4% paraformaldehyde for 30 min at room temperature and then blocked with 5% bovine serum albumin (BSA) (Yeasen Biotechnology, Shanghai, China) for 1 h at room temperature. For detection of N protein expression, cells were incubated with an anti‐N protein rabbit monoclonal antibody (1:5000; GeneTex, San Antonio, TX, USA) for 1 h at 37°C. After washing, the cells were stained with FITC‐conjugated goat anti‐rabbit IgG. Nuclei were counterstained with 4′,6‐diamidino‐2‐phenylindole (DAPI) solution (Solarbio Life Sciences, Beijing, China) for 5 min at room temperature. After thorough rinsing with 1× phosphate‐buffered saline (PBS), fluorescent images were captured using an epifluorescence microscope.

### 2.4. Full‐Length Infectious Clone Construction of JS‐YZ/2023

The full‐length infectious clone of JS‐YZ/2023 was constructed as follows. MARC‐145 cells infected with JS‐YZ/2023 at an MOI of 0.01 were harvested upon observation of CPE and subjected to three freeze–thaw cycles. Viral RNA was extracted using AG RNAex Pro reagent (Accurate Biology, Hangzhou, China), and first‐strand cDNA was synthesized with M‐MLV reverse transcriptase. The complete JS‐YZ/2023 sequence, determined by full‐length sequencing, was amplified from cDNA using specific primers listed in Table 1. Six overlapping genomic segments were assembled into three contiguous fragments by gene splicing. The pACYC177‐CMV vector was linearized by double restriction enzyme digestion, and a homologous recombination kit was used to directionally insert the three viral fragments into the linearized vector, as shown in Figure 1A. The corresponding nucleic acid gel electrophoresis results are shown in Figure 1C. The assembled full‐length cDNA clone, designated pACYC177‐JS‐YZ/2023, was sequence‐verified.

Figure 1Construction of the JS‐YZ/2023 reverse genetics system. (A) Full‐length cDNA clone. A three‐step cloning strategy using in vitro homologous recombination generated the full‐length cDNA clone pACYC177‐JS‐YZ/2023 containing a genetic marker. The viral genome was divided into six fragments. First, a shuttle plasmid (pACYC177‐CMV) was constructed by inserting the CMV promoter, a SwaI site, and the HDV ribozyme into pACYC177. Next, adjacent fragments were fused to create three intermediate vectors (pTOPO‐4622bp, pTOPO‐7022bp, and pTOPO‐4558bp) via zero‐background cloning. Finally, these fragments were digested and assembled with pACYC177‐CMV using multifragment homologous recombination. (B) GFP‐expressing clone. A GFP cassette was designed with partial ORF1b upstream and TRS6 plus partial ORF2 downstream, preserving the *Asc*I and *Eco*RV sites. The full‐length clone and GFP fragment were digested and ligated using T4 DNA ligase, yielding pACYC177‐GFP‐JS‐YZ/2023. (C) Electrophoresis analysis of PCR‐amplified fragments (F1–F6), positive clones, linearized templates, and in vitro–transcribed RNA. M1, M2, and M3: markers; fragment 1 + 2 = 4622 bp; fragment 3 + 4 = 7022 bp; fragment 5 + 6 = 4558 bp; *Sbf*I‐pACYC177‐CMV‐*Mre*I = 3522 bp; GFP = 720 bp; *Asc*I–GFP = 318 bp; ORF2a–*Eco*RV = 817 bp; *Asc*I–GFP–*Eco*RV = 1136 bp; *Asc*I–pACYC177–JS‐YZ/2023–*Eco*RV = 19591 bp; *Nde*I–pACYC177–GFP–JS‐YZ/2023–*Mre*I = 16,615 and 3173 bp.

**Figure:**
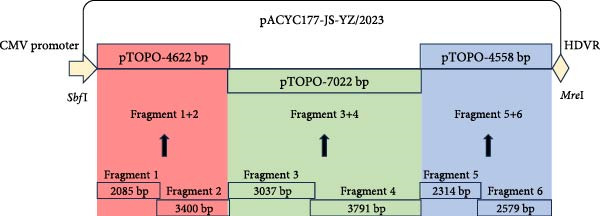
(A)

**Figure:**
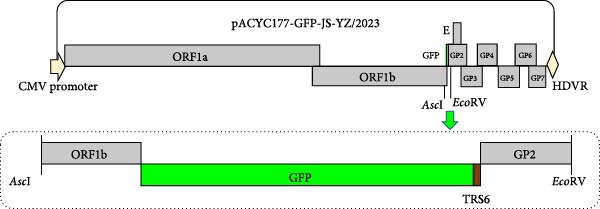
(B)

**Figure:**
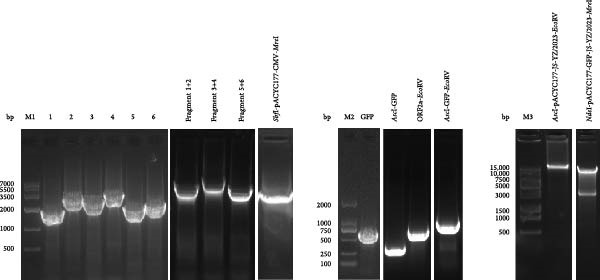
(C)

**Table 1:** Primers used for PCR Assay.

Primer name	Primer sequence(5′‐3′)	Fragment length (bp)
PRRSV‐1F	ATGACGTATAGGTGTTGG	2085
PRRSV‐1R	CAGTCCACACAACAGG	

PRRSV‐2F	CAACTTTGGTCGTTCGACC	3400
PRRSV‐2R	CGACCACATTGACGG	

PRRSV‐3F	GAGGGCGCTTTCAGAACT	3037
PRRSV‐3R	CCTGTGCGCACAGAA	

PRRSV‐4F	CATTCAGAGCGTCCCTG	3791
PRRSV‐4R	CCACAAAAAGTTGGCC	

PRRSV‐5F	GCCCTGTCATTGAACCAACTTTAGGC	2314
PRRSV‐5R	CGCTGAAATTTTGGTTACGGGGG	

PRRSV‐6F	CTTGATGGTTCCGCGGCAA	2579
PRRSV‐6R	GGGACCATGCCGGCC	

GFP‐F	GAATTGAAATGGTGAGCAAGGGCGAG	744
GFP‐R	GGTTGCCGCGGAACTCACTTGTACAGCTCG	

Partial ORF1b‐F	AAACACACCTGGGGA	207
Partial ORF1b‐R	AACAGCTCCTCGCCCTTGCTCACCATTTCAATTCAGGCC	

Partial ORF2‐F	AGTTCCGCGGCAACCCCTTTAACCAGAGTTTCAGCGGAACTATGAAATGGGGTCTATGC	130
Partial ORF2‐R	ATCAACAATGGACACCAG	

### 2.5. Generation of JS‐YZ/2023 Infectious Clone Plasmid Incorporating the GFP Gene

To generate a fluorescent reporter PRRSV, the GFP coding sequence was inserted into the JS‐YZ/2023 genome between ORF1b and ORF2. Partial ORF1b was placed upstream of GFP, while TRS6 and partial ORF2 were placed downstream, preserving the *Asc*I and *EcoR*V sites. The plasmid pACYC177‐JS‐YZ/2023 and the GFP fusion fragment were digested with the corresponding restriction enzymes, and ligation was performed using T4 DNA ligase (New England Biolabs, Ipswich, MA, USA). The resulting infectious clone, designated pACYC177‐GFP‐JS‐YZ/2023, enabled stable GFP expression (Figure 1B). The corresponding nucleic acid gel electrophoresis results are shown in Figure 1C.

### 2.6. Virus Rescue

BHK‐21 cells at approximately 80% confluency in 24‐well plates were transfected with 1 μg of full‐length cDNA plasmid (pACYC177‐JS‐YZ/2023 or pACYC177‐GFP‐JS‐YZ/2023) using TurboFect reagent (Thermo Fisher Scientific), following the manufacturer’s protocol. Supernatants collected at 24 hpt were inoculated onto MARC‐145 cells seeded 24 h earlier. CPE and GFP expression were monitored daily to confirm viral rescue. Supernatants were harvested at approximately 4 dpi, when significant CPE was observed. These constituted the F1 virus stocks, designated rJS‐YZ/2023 and rGFP‐JS‐YZ/2023, and were stored at −80°C.

### 2.7. Western Blotting Assay

The harvested viral supernatant was transferred onto MARC‐145 cells in 12‐well plates. After 24 hpi, the supernatant was removed, and the cells were gently rinsed once with PBS. Subsequently, 1 mL of PBS was added to the wells, and the cells were scraped for collection. Equivalent amounts of total protein were separated by sodium dodecyl sulfate–polyacrylamide gel electrophoresis and transferred onto Amersham Protran Premium 0.2 NC nitrocellulose membranes (Cytiva, Danaher Corporation, Washington, D.C., USA). Membranes were blocked in Tris‐buffered saline with Tween 20 containing 5% BSA for 2 h at room temperature, then incubated with primary antibodies against β‐actin (1:10,000; Proteintech, Wuhan, China) and PRRSV‐N at 4°C overnight. After washing, membranes were incubated with horseradish peroxidase‐conjugated goat anti‐rabbit IgG for 2 h at room temperature. Immunoreactive proteins were visualized using an enhanced chemiluminescence detection kit (CWBIO, Beijing, China).

### 2.8. Viral Growth Kinetics Assessment

To evaluate viral growth kinetics, MARC‐145 cells in 24‐well plates were inoculated at an MOI of 0.01. After a 2‐h adsorption period at 37°C, the cells were washed three times with PBS following removal of the inoculum, and fresh maintenance medium was added. Supernatants were harvested at 12, 24, 36, 48, 60, 72, 84, and 96 hpi. Viral titers were quantified using the 50% tissue culture infectious dose (TCID_50_) method.

### 2.9. Sequence Analysis

Nucleotide homology was assessed using MEGA 7, and GenBank reference sequences were aligned accordingly (Table 2). Phylogenetic trees of diverse PRRSV lineages were constructed in MEGA 11 based on Nsp2 and ORF5 gene sequences, aligned with ClustalW algorithms. Analyses were performed with 1000 bootstrap replicates. Recombination between the JS‐YZ/2023 isolate and reference PRRSV strains was examined using SimPlot software with a 200‐bp sliding window moving in 20‐bp steps across genome alignments. Potential N‐glycosylation sites within the GP5 protein were predicted using the NetNGlyc 1.0 Server: NetNGlyc‐1.0‐redirect (dtu.dk) [18].

**Table 2:** The 34 representative PRRSV strains used for sequence comparative analysis.

No.	Strains	GenBank No.	Genealogy	Reported date
1	FJ1402	KX169191.1	L1	23 November 2016
2	FJY04	KP860910.1	L1	19 August 2015
3	FJZ03	KP860909.1	L1	19 August 2015
4	HENAN‐XINX	KF611905.1	L1	14 July 2015
5	CHsx1401	KP861625.1	L1	17 December 2015
6	NADC30	JN654459.1	L1	12 October 2012
7	HNjz15	KT945017.1	L1	03 May 2016
8	15SC3	KX815428.1	L1	17 April 2017
9	HENAN‐HEB	KJ143621.1	L1	02 April 2014
10	15LN3	KX815425.1	L1	17 April 2017
11	15HEN1	KX815413.1	L1	17 April 2017
12	HENZMD‐9	KU950374.1	L1	25 May 2016
13	SC‐d	MF375261.1	L1	20 May 2018
14	CHNMGKL1‐2304	OR753369.1	L1	13 November 2023
15	JS2021NADC34	MZ820388.1	L1	18 January 2022
16	LNDZD10‐1806	MN648054.1	L1	25 February 2020
17	HLJTZJ829‐2010	OL516349.1	L1	21 March 2022
18	IA/2014/NADC34	MF326985.1	L1	02 December 2017
19	JL580	KR706343.1	L1	23 August 2015
20	GM2	JN662424.1	L3	13 August 2012
21	QYYZ	JQ308798.1	L3	13 August 2012
22	VR2332	EF536003.1	L5	30 May 2014
23	CH‐1a	AY032626.1	L8	22 July 2016
24	CH‐1R	EU807840.1	L8	26 July 2016
25	SD‐YL1712	MT708500.1	L8	09 November 2020
26	TJnh1501	KX510269.1	L8	07 May 2017
27	JXA1	EF112445.1	L8	14 July 2016
28	TJbd14‐1	KP742986.1	L8	31 January 2016
29	09HEN1	JF268684.1	L8	25 July 2016
30	WUH3	HM853673.2	L8	12 March 2013
31	GD‐HD	KP793736.1	L8	10 August 2015
32	TJ	EU860248.1	L8	25 April 2012
33	HUN4	EF635006.1	L8	23 July 2016
34	JXwn06	EF641008.1	L8	23 July 2016

### 2.10. Animal Studies for Pathogenicity Evaluation

To evaluate the pathogenicity of JS‐YZ/2023, six 1‐month‐old PRRSV antigen‐/antibody‐negative piglets were selected in compliance with the 3R principle. The piglets were randomly assigned to either an inoculation group or a control group, with three animals per group. The inoculation group received 2 × 10^5^ TCID_50_ of JS‐YZ/2023 via intramuscular injection and 1 × 10^5^ TCID_50_ via intranasal drops, while the control group received equivalent volumes of DMEM through the same routes. All piglets were housed individually with ad libitum access to food and water. Postinoculation, body temperature, weight, feed intake, and clinical signs were recorded daily. Blood samples were collected every other day and analyzed for PRRSV antibodies using a PRRSV antibody enzyme‐linked immunosorbent assay (ELISA) kit (JNT, Beijing, China). Viral shedding and viremia were assessed by quantifying viral copy numbers in swabs and blood using reverse‐transcription quantitative polymerase chain reaction (RT‐qPCR). On day 21 postinoculation, the piglets were euthanized. Necropsy included collection of lung, ileal tissue (10–15 cm anterior to the ileocecal junction), heart, liver, spleen, kidney, submandibular lymph node, thymic lymph node, and inguinal lymph node. Ileal and lung samples were processed for histopathological examination by fixation in paraformaldehyde, paraffin embedding, sectioning, and hematoxylin and eosin (H&E) staining, as well as immunohistochemical analysis. Viral loads in all collected tissues were quantified using RT‐qPCR.

### 2.11. Cytokine Quantification

Tissue samples (≥100 mg) were aseptically collected and homogenized at 21 dpi. Total RNA was extracted from homogenates using TRIzol Reagent (Invitrogen, Thermo Fisher Scientific). cDNA was synthesized with the PrimeScript RT reagent kit (Takara Bio, Beijing, China). qPCR was performed using TB Green Premix Ex Taq (Takara Bio) on a QuantStudio 5 system (Applied Biosystems, Thermo Fisher Scientific). Gene expression was normalized to β‐2‐microglobulin and calculated using the 2^−ΔΔCt^ method.

### 2.12. Statistical Analysis

All experiments were performed in triplicate with independent replicates. Data were analyzed using GraphPad Prism Software (v8.0.2). Results are presented as mean ± standard deviation. Statistical significance between two groups was determined with a two‐tailed unpaired *t*‐test, while comparisons among three or more groups were assessed by one‐way analysis of variance with multiple comparisons. A *p*‐value of <0.05 was considered statistically significant.

## 3. Results

### 3.1. Virus Isolation

**Figure 2:**
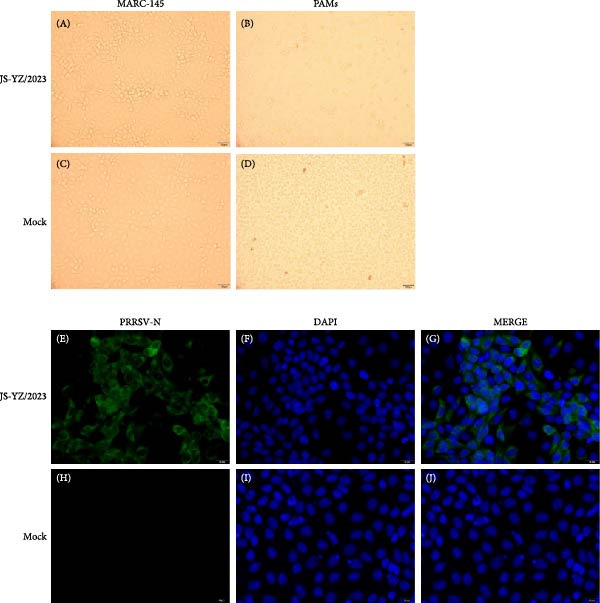
CPEs induced by the JS‐YZ/2023 strain. (A) MARC‐145 cells and (B) porcine alveolar macrophages showed characteristic lesions. (E–G) Indirect immunofluorescence assay results in JS‐YZ/2023–infected cells. Corresponding mock‐infected controls are shown for (C, D) lesion observation and (H–J) immunofluorescence assay.

From a PRRS‐suspected pig farm in Jiangsu Province, we isolated a PRRSV strain designated JS‐YZ/2023 (GenBank Accession Number PX136374). Filtered tissue homogenates were inoculated onto MARC‐145 cells. After three blind passages, characteristic grape‐like cluster CPE appeared in MARC‐145 cells. Supernatants harvested from these cultures induced significant cytopathology in PAMs (Figure 2A–D). Virus isolation was confirmed by TCID_50_ assay and immunofluorescence assay targeting the PRRSV‐N protein. MARC‐145 cells infected with JS‐YZ/2023 at an MOI of 0.01 exhibited strong N protein expression at 24 hpi (Figure 2E–G), confirming successful viral replication and protein synthesis.

### 3.2. Rescue Viruses

**Figure 3:**
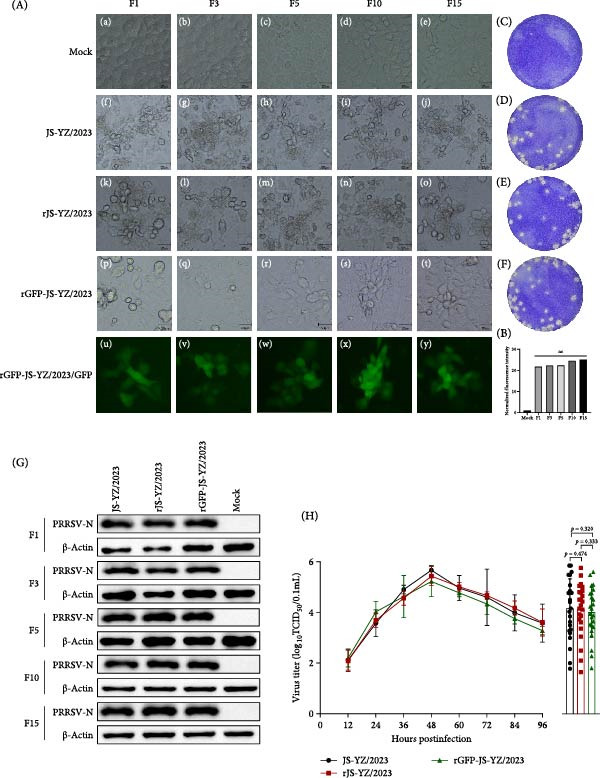
Characteristics of JS‐YZ/2023, rJS‐YZ/2023, and rGFP‐JS‐YZ/2023. (A) Continuous passage of JS‐YZ/2023, rJS‐YZ/2023, and rGFP‐JS‐YZ/2023 in MARC‐145 cells up to passage 15 (F15). (a–e) Mock group; (f–j) JS‐YZ/2023; (k–o) rJS‐YZ/2023; (p–t) rGFP‐JS‐YZ/2023; (u–y) rGFP‐JS‐YZ/2023 showing GFP expression. (B) Average fluorescence intensity of (u–y) analyzed using ImageJ. (C–F) Plaque morphology at 4 dpi (crystal violet staining): (C) mock, (D) JS‐YZ/2023, (E) rJS‐YZ/2023, (F) rGFP‐JS‐YZ/2023 (MOI = 0.01). (G) Western blot analysis of PRRSV‐N protein expression in infected cell supernatants. (H) Growth kinetics in MARC‐145 cells (MOI = 0.01). Viral titers in supernatants were measured every 12 h (mean ± SD).

BHK‐21 cells transfected with the two infectious clones produced rescued viruses (rJS‐YZ/2023 and rGFP‐JS‐YZ/2023). Supernatants harvested at 24 hpt were used to infect MARC‐145 cells, where CPE appeared by 48 hpi. Both rescued viruses exhibited biological properties comparable to the parental strain JS‐YZ/2023. After 15 serial passages, rGFP‐JS‐YZ/2023 maintained stable GFP expression intensity (Figure 3A,B). Western blot analysis of passaged cells confirmed consistent PRRSV‐N protein expression (Figure 3G). To further evaluate viral characteristics, plaque morphology and multi‐step growth curves were examined in MARC‐145 cells as previously described [19, 20]. Viral plaques showed identical morphology across all strains (Figure 3C–F). Growth kinetics revealed peak titers of 10^5.88^ TCID_50_/0.1 mL at 48 hpi, with rescued viruses displaying replication dynamics indistinguishable from JS‐YZ/2023 (Figure 3H). These results demonstrate the successful rescue of a genetically stable GFP‐expressing PRRSV.

### 3.3. Recombinant Analysis

The complete genome of JS‐YZ/2023 (15,010 nt) was assembled using DNAStar SeqMan. Full genome comparisons revealed that JS‐YZ/2023 shared the highest identity (92.4%) with HP‐PRRSV (Lineage 8.3), while identity with NADC30 (Lineage 1.8) was 88.5%. Homology analysis showed NADC30‐like similarity in ORF1a (90.0%), ORF5 (95.4%), and ORF6 (97.5%), but HP‐PRRSV‐like identity in ORF1b–ORF4 and ORF7 (Figure 4A).

Figure 4Genomic analysis of JS‐YZ/2023. (A) Nucleotide sequence comparison of JS‐YZ/2023 with the prototype PRRSV‐2 strain. (B) Recombination analysis (SimPlot v3.5.1) using parental references (JN654459), (EF112445), CH‐1a (AY032626.1), and IA/2014/ (MF326985.1). Parameters: window size, 200 bp; step size, 20 bp. *Y*‐axis indicates nucleotide similarity. (C) Schematic diagram of the JS‐YZ/2023 genome with annotated protein‐coding regions. (D) Phylogenetic trees (MEGA7; neighbor‐joining method; 1000 bootstrap replicates) reconstructed from SimPlot‐defined genomic segments: (D1) Segment a; (D2) Segment b; (D3) Segment c; (D4) Segment d.

**Figure:**
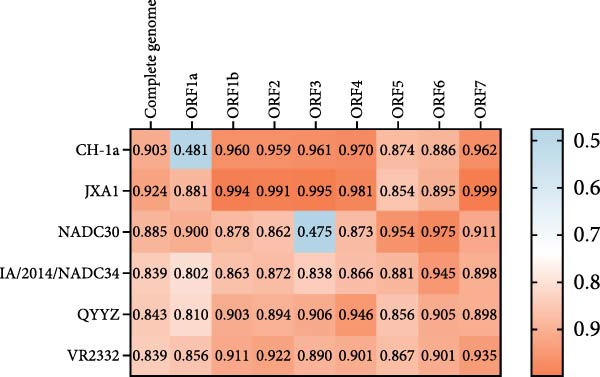
(A)

**Figure:**
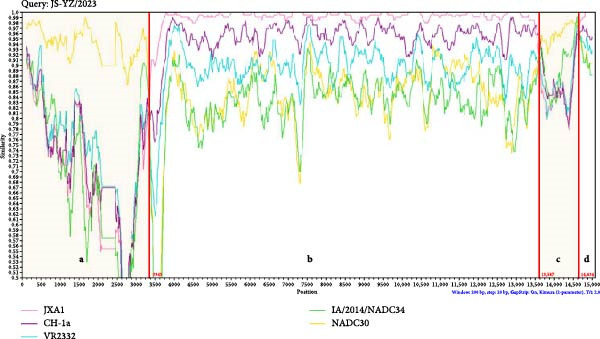
(B)

**Figure:**
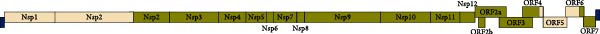
(C)

**Figure:**
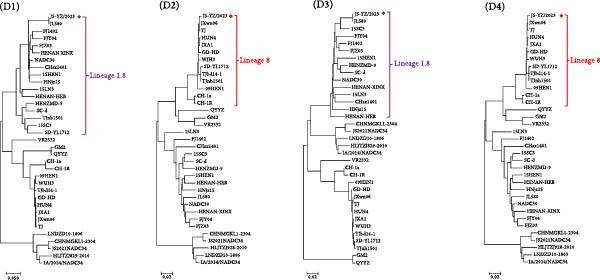
(D)

Similarity analysis was performed against classical strains VR2332, NADC30, JXA1, CH‐1a, and IA/2014/NADC34. Recombination analysis identified breakpoints at nt 3343, 13,587, and 14,634, dividing the genome into four segments (Figure 4B). Segments (a) and (c) originated from NADC30‐like strains, whereas segments (b) and (d) were derived from HP‐PRRSV. The recombinant regions spanned NSP1, partial NSP2, minor GP4, full GP5, and major GP6 (Figure 4C).

To further elucidate the genetic evolution of JS‐YZ/2023, a phylogenetic tree was reconstructed using four genomic fragments from 34 reference strains. Phylogenetic analysis of segment (b) confirmed clustering with Lineage 8 (HP‐PRRSV), consistent with segment (d), while segments (a) and (c) grouped with Lineage 1.8 (NADC30‐like) (Figure 4D).

### 3.4. Nsp2 and GP5 Amino Acid Analysis

Nsp2, a PRRSV protein with a high mutation rate and a key driver of viral evolution, exhibited a discontinuous 111 + 1 + 19 amino acid deletion in the JS‐YZ/2023 strain (Figure 5A). This deletion pattern is consistent with that of NADC30‐like reference strains. Comparative analysis of GP5 revealed a conserved protein length but elevated amino acid substitutions in JS‐YZ/2023 (Figure 5B). These variations mapped predominantly within T‐cell and B‐cell epitopes across conserved domains, suggesting potential roles in immune evasion. Sublineage‐specific differences were also observed, with JS‐YZ/2023 (Lineage 1.8) carrying unique epitope mutations C/Y10, S/D/Y/N61, and I/V121. In addition, GP5 of JS‐YZ/2023 was predicted to contain four N‐glycosylation sites (Table 3).

Figure 5Amino acid sequence alignment of Nsp2 and GP5 in JS‐YZ/2023 compared with reference PRRSV‐2 strains. (A) Alignment of Nsp2. Deleted regions are boxed, corresponding to positions 322–432, 481, and 527–546 in the NADC30 Nsp2 protein. (B) GP5 analysis. Gray boxes indicate the signal peptide and transmembrane (TM) regions. Yellow, green, and blue shading mark the decoy epitope, hypervariable region (HVR), and PNE linear antigenic epitope, respectively. Red and purple boxes denote B‐cell and T‐cell epitopes.

**Figure:**
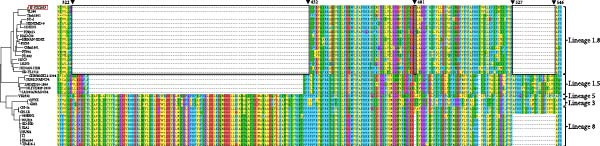
(A)

**Figure:**
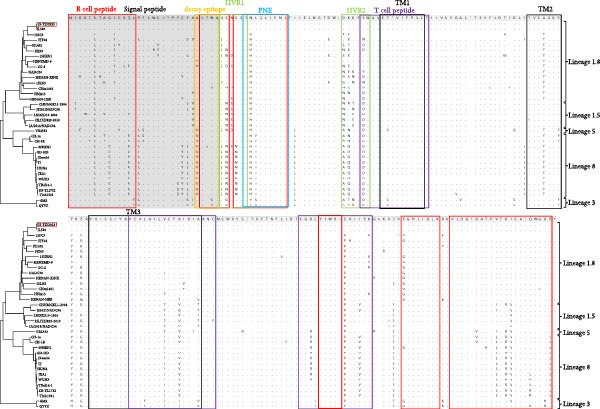
(B)

**Table 3:** NGSs number analysis.

Isolates	Lineage	N‐glycosylation sites					NGSs number
		N30	N32/N33/N34	N35	N44	N51	
JS‐YZ/2023	1.8	√	√	—	√	√	4
NADC30	1.8	√	√	—	√	√	4
SC‐d	1.8	√	—	—	√	√	3
SD‐YL1712	1.8	√	√	√	√	√	5
TJnh1501	1.8	√	√	√	√	√	5
15HEN1	1.8	√	√	—	√	√	4
15LN3	1.8	√	√	—	√	√	4
15SC3	1.8	√	√	—	√	√	4
CHsx1401	1.8	—	√	—	√	√	3
FJ1402	1.8	√	√	—	√	√	4
FJY04	1.8	√	√	—	√	√	4
FJZ03	1.8	√	√	—	√	√	4
HENAN‐HEB	1.8	√	√	—	√	√	4
HENAN‐XINX	1.8	√	√	—	√	√	4
HENZMD‐9	1.8	√	√	—	√	√	4
HNjz15	1.8	√	√	—	√	√	4
JL580	1.8	√	√	—	√	√	4
JS2021NADC34	1.5	√	√	—	√	√	4
LNDZD10‐1806	1.5	√	√	—	√	√	4
IA/2014/NADC34	1.5	√	√	—	√	√	4
CHNMGKL1‐2304	1.5	√	√	—	√	√	4
HLJTZJ829‐2010	1.5	√	√	—	√	√	4
QYYZ	3	—	√	—	√	√	3
GM2	3	—	√	—	√	√	3
VR2332	5.1	√	√	—	√	√	4
JXA1	8.7	√	√	√	√	√	5
JXwn06	8.7	√	√	√	√	√	5
TJ	8.7	√	√	√	√	√	5
TJbd14‐1	8.7	√	√	√	√	√	5
WUH3	8.7	√	√	√	√	√	5
09HEN1	8.7	√	√	√	√	√	5
CH‐1a	8.7	√	√	—	√	√	4
CH‐1R	8.7	√	√	—	√	√	4
GD‐HD	8.7	√	√	√	√	√	5
HUN4	8.7	√	√	√	√	√	5

### 3.5. Pathogenicity Assessment in Piglets

A pathogenicity study was conducted in 1‐month‐old piglets inoculated with the JS‐YZ/2023 strain via intramuscular injection and intranasal administration. Challenged piglets developed marked anorexia, diarrhea, lethargy, and reduced weight gain compared with controls (Figure 6A,B). Two piglets exhibited fever, peaking at 40.8°C at 1 dpi (Figure 6C). Viral shedding was detected in nasal, oral, and anal swabs, with anal viral loads significantly exceeding those of other samples between 3 and 11 dpi (Figure 6D–G). Viremia appeared at 1 dpi, peaked at 10^7.19^ copies/mL by 11 dpi, and persisted throughout the trial (Figure 6H). Seroconversion occurred at 9 dpi, with antibody levels remaining high thereafter (Figure 6I).

Figure 6Clinical signs and virological parameters in piglets infected with JS‐YZ/2023. (A) Clinical scores. (B) Average daily weight gain rate. (C) Rectal temperature (fever defined as >40.0°C). (D–F) Viral genome copies (qPCR) in nasal, oral, and anal swabs. (G) Comparison of viral loads across swabs. (H, J) Viral genome copies (qPCR) in blood and tissues. (I) Serum N protein antibody titers measured by ELISA (S/*p*  > 0.4 indicates seroconversion). Data represent mean ± SD, analyzed using GraphPad Prism v8).  ^∗^ *p*  < 0.05;  ^∗∗^ *p*  < 0.01;  ^∗∗∗^ *p*  < 0.001.

**Figure:**
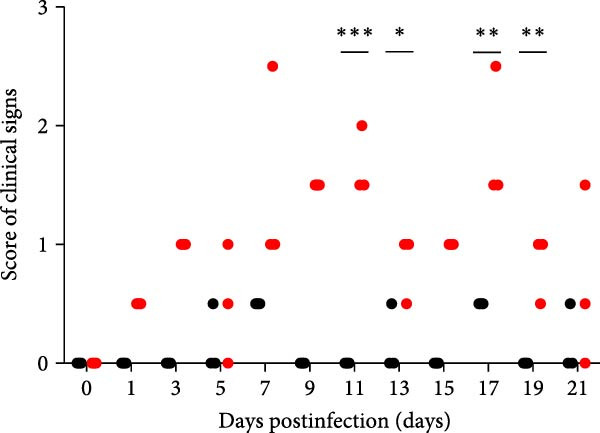
(A)

**Figure:**
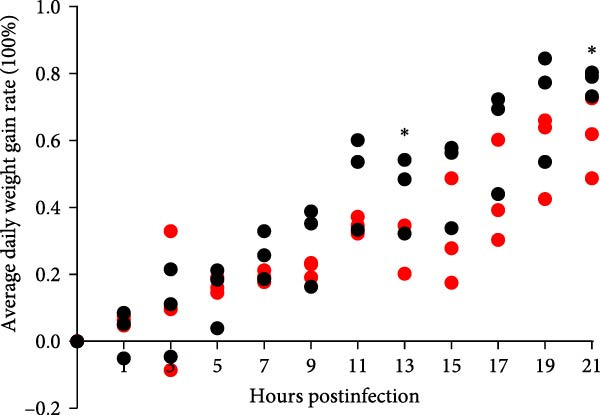
(B)

**Figure:**
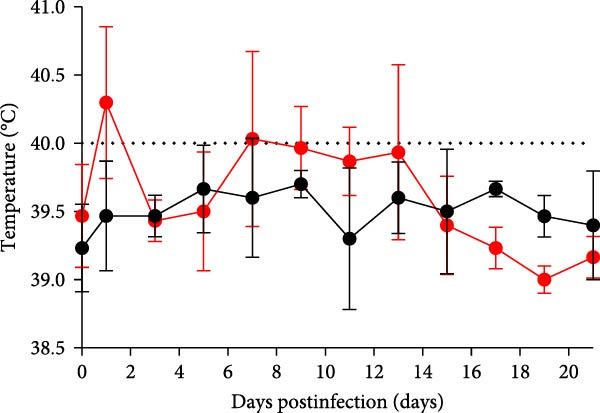
(C)

**Figure:**
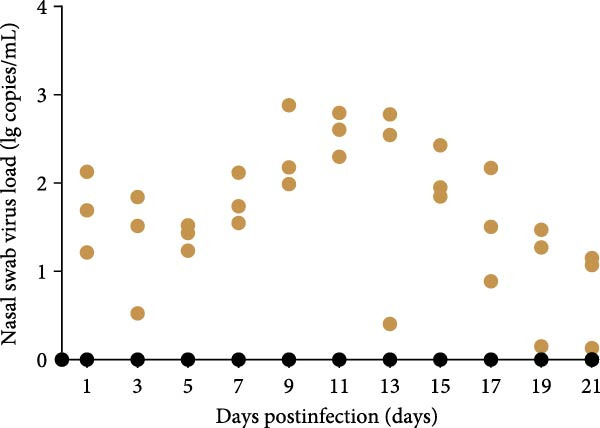
(D)

**Figure:**
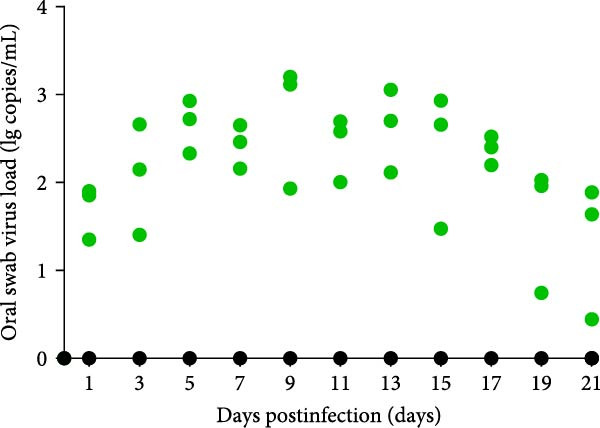
(E)

**Figure:**
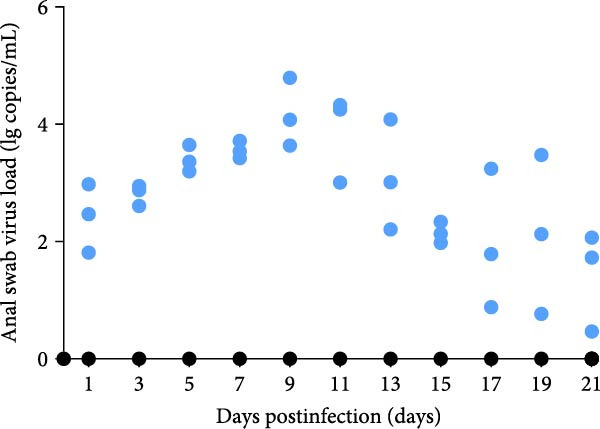
(F)

**Figure:**
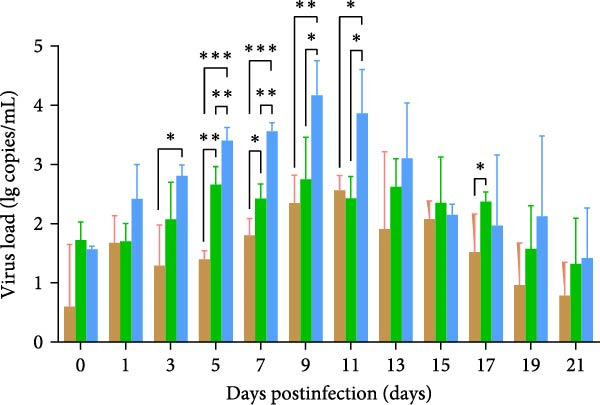
(G)

**Figure:**
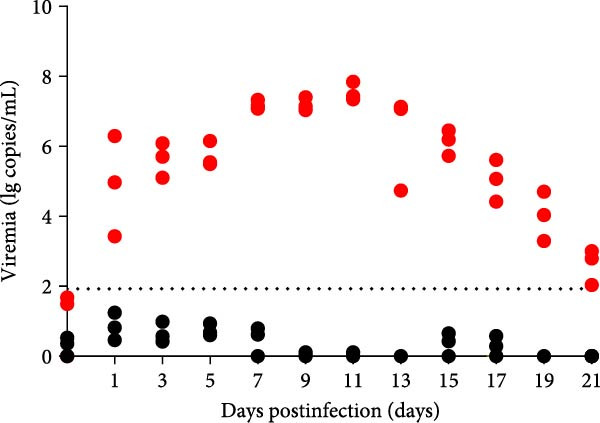
(H)

**Figure:**
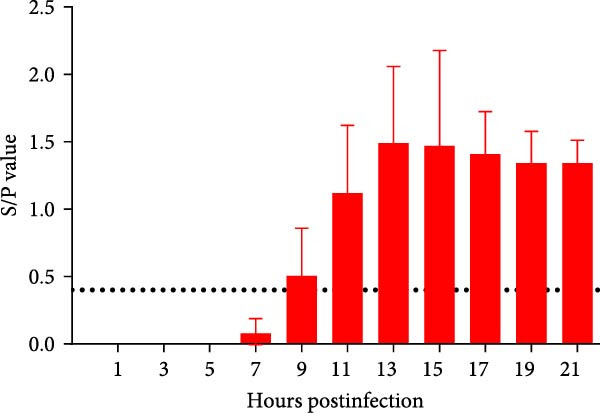
(I)

**Figure:**
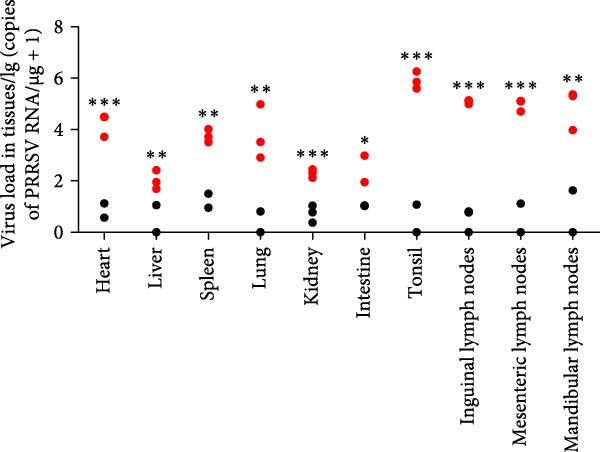
(J)

**Figure 7:**
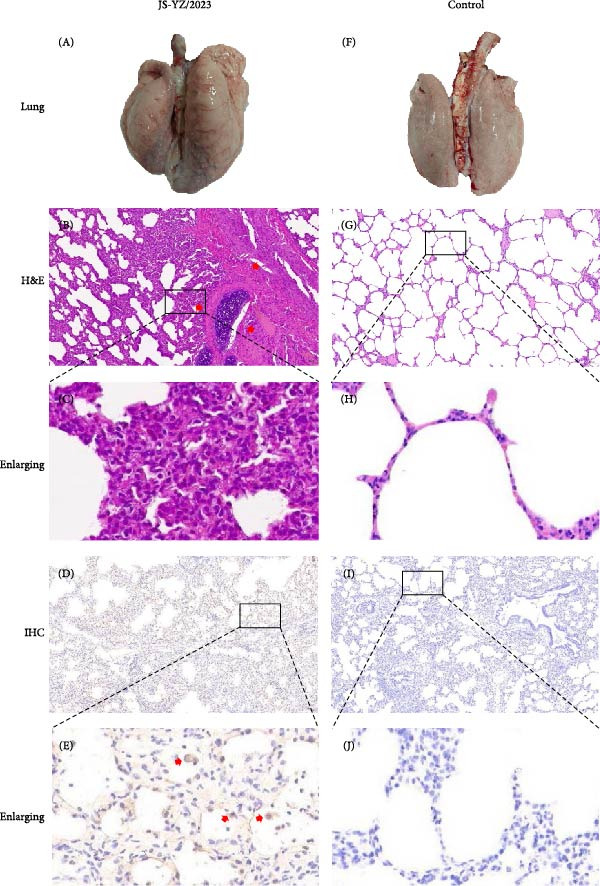
Pathological lung lesions in JS‐YZ/2023–infected piglets. Gross, histological, and immunohistochemical findings in lungs from infected and control piglets. Consolidation and ecchymosis in (A) infected lungs compared with (F) controls. Extensive serous and interstitial pneumonia in (B) infected lungs compared with (G) controls. Positive brown–red macrophages in (D) infected lungs compared with (I) controls. (C, E, H, J) Enlarged insets highlighting areas from (B), (D), (G), and (I), respectively. Scale bars = 50 μm. (F–J) Control group: normal alveolar structure.

**Figure 8:**
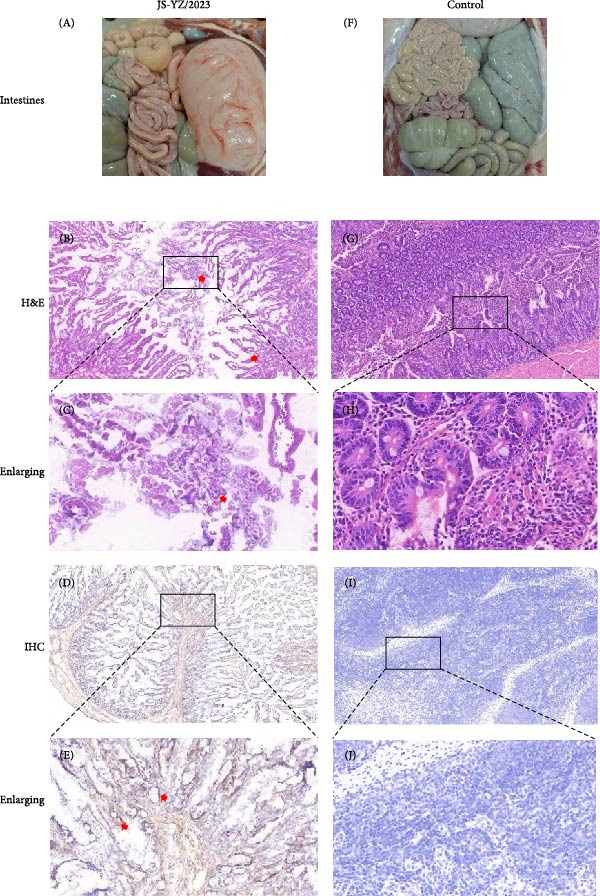
Histopathological examination of the ileum in piglets. (A) Thinning of the intestinal wall in virus‐challenged piglets, with dehydration and loss of elasticity. (B) Microscopic lesions (H&E): inflammatory cell infiltration; shortened, thickened, and partially fused villi (infected group). Arrows highlight lesions. (D) Immunohistochemistry: JS‐YZ/2023 antigen (brown) clustered near crypt glands (infected group). Arrows highlight positive signals. (F–J) Control group: normal intestinal morphology. (C, E, H, and J) Enlarged insets highlighting areas from (B), (D), (G), and (I), respectively. Scale bars = 50 μm.

Postmortem examination at 21 dpi revealed systemic infection, with the highest viral loads in tonsils and lymph nodes (Figure 6J). Lung pathology included severe interstitial pneumonia characterized by alveolar septal thickening and inflammatory infiltration (Figure 7A–C). Immunohistochemistry using a monoclonal antibody specific to PRRSV nucleocapsid protein revealed viral antigen in lung tissues, with positive brown –red macrophages primarily showing cytoplasmic staining and occasional granular distribution (Figure 7D,E). Intestinal lesions included villus shortening and mucosal disintegration (Figure 8A–C), with PRRSV antigen detected in crypt epithelial cells (Figure 8D,E). Control piglets showed no clinical symptoms, remained virus free, and exhibited no pathological changes throughout the experiment (Figures 6– 8, control panels).

### 3.6. Cytokine Concentrations in Tissues

Post challenge immune responses in piglets were evaluated by quantifying inflammatory factor concentrations across tissues. Expression levels, normalized to β‐2‐microglobulin, revealed significant tissue‐specific variation (Figure 9). From Figure 9A–F were TNF‐α, IL‐6, IL‐8, IL‐10, IFN‐α, and IFN‐γ. The strongest immune responses were observed in the intestine and mesenteric lymph nodes, while responses in the heart and kidney were comparatively weak. The occurrence of diarrhea in challenged piglets indicated that the intestine served as a primary target site for JS‐YZ/2023, driving robust antiviral responses. Elevated interferon (IFN) concentrations in mesenteric lymph nodes further reflected active antiviral and cell‐mediated immunity within this tissue.

Figure 9Relative mRNA expression levels of cytokines in tissue homogenates from piglets infected with JS‐YZ/2023 at 21 dpi. Cytokine levels were measured by relative fluorescence quantification and normalized to β‐2‐microglobulin. (A) TNF‐α, (B) IL‐6, (C) IL‐8, (D) IL‐10, (E) IFN‐α, and (F) IFN‐γ. Data are presented as mean ± SD.

**Figure:**
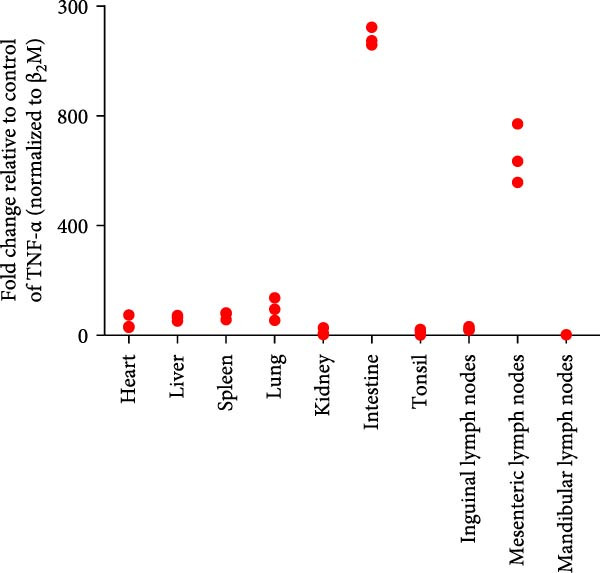
(A)

**Figure:**
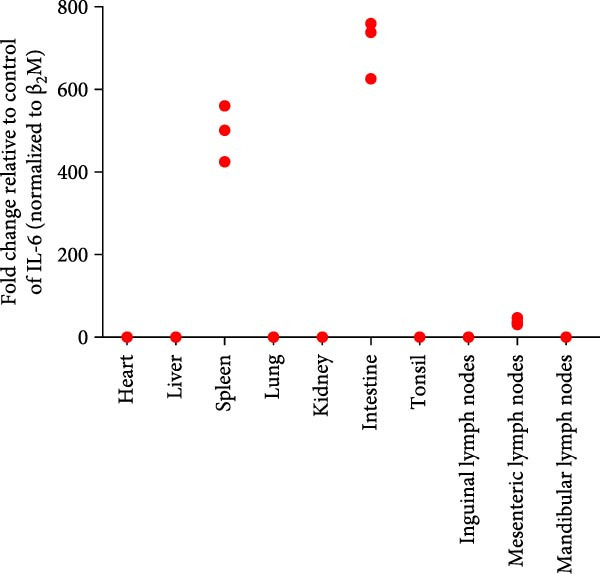
(B)

**Figure:**
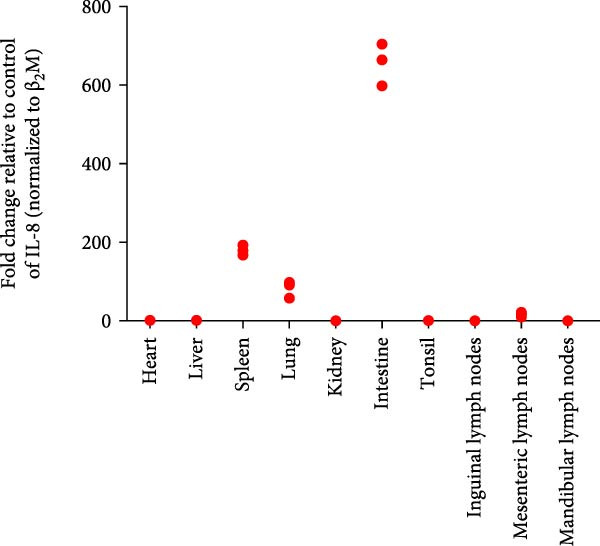
(C)

**Figure:**
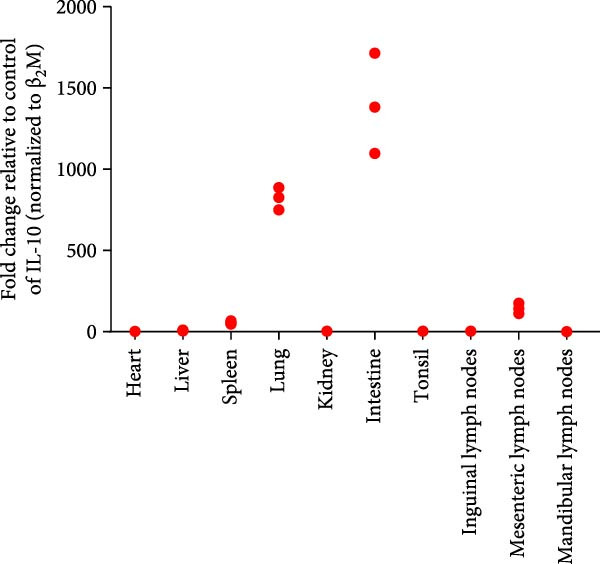
(D)

**Figure:**
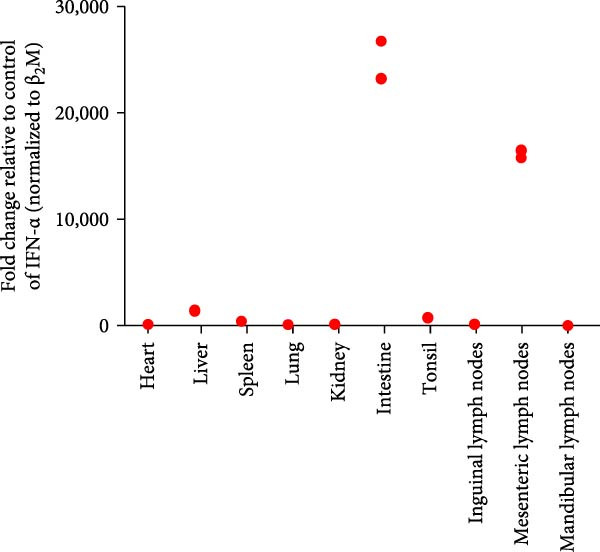
(E)

**Figure:**
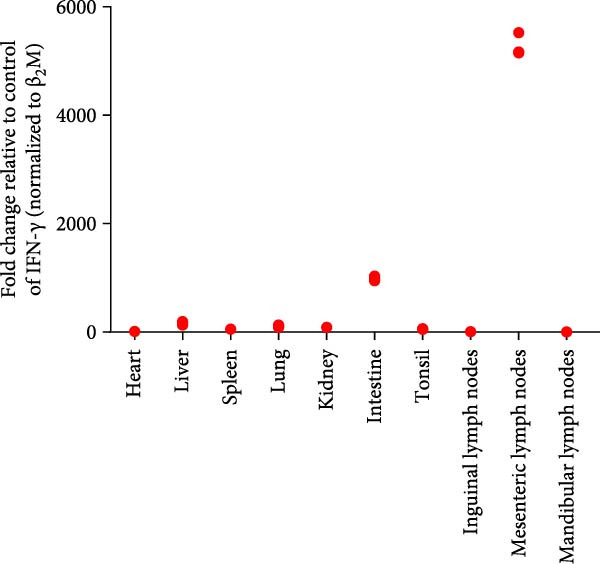
(F)

## 4. Discussion

PRRSV, first identified in China in 1996 with strain CH‐1a [21], has since become one of the country’s most widespread swine diseases. Since 2013, NADC30‐like PRRSV strains have emerged in China, showing extensive recombination and dissemination [22]. These variants have contributed to suboptimal cross‐protection by existing vaccines [23], and their prevalence has continued to rise since 2014 across multiple provinces, including Beijing, Tianjin, Hebei, Shandong, and Guangdong. Genetic classification of PRRSV‐2 relies primarily on ORF5 gene analysis for three main reasons. First, ORF5 encodes the major envelope glycoprotein GP5, which contains frequently mutating neutralizing and decoy epitopes, providing strong phylogenetic signal. Second, refined classification based on more than 82,000 global sequences has defined 11 major lineages and 21 sublineages, allowing consistent tracking of strains such as NADC30‐like (Lineage 1) [24]. Third, ORF5 sequences correlate with viral pathogenicity and vaccine efficacy. Recent advances in next‐generation computational tools now automate lineage calling with high accuracy, reinforcing ORF5’s role in molecular surveillance [25]. While whole genome analysis remains indispensable for recombination studies, ORF5 phylogeny continues to serve as the gold standard for initial lineage assignment.

Recombination is highly prevalent among PRRSV isolates in China, particularly within NADC30‐like lineages [26]. Surveillance has revealed recombination events not only between HP‐PRRSV and NADC30‐like strains but also among NADC30‐like, VR‐2332‐like, and CH‐1a‐like variants [27–31]. This recombination, driven by the coexistence of multiple strains, generates diverse recombinant viruses that increase genomic diversity and complicate disease control [32]. While the recombination rate remained relatively low from 2006 to 2011 [33], the emergence of NADC30‐like strains dramatically increased the frequency of detected recombinants [34]. Analyses of these strains consistently identify recombination hotspots in Nsp1, Nsp4–9, and ORF2–ORF4, with NADC30‐like strains frequently serving as the main parental lineage [27].

The isolated strain JS‐YZ/2023 is a NADC30‐like recombinant, with HP‐PRRSV as the major parental strain and a NADC30‐like strain as the donor. The recombination regions spanned Nsp1–Nsp2 and ORF4–ORF6 (Figure 4C). Despite its HP‐PRRSV parentage, the Nsp2 gene of JS‐YZ/2023 exhibits the characteristic NADC30‐like 111 + 1 + 19 amino acid deletion [35] (Figure 5A). Whole genome alignment showed the highest identity with HP‐PRRSV, whereas phylogenetic analyses of Nsp2 and ORF5 confirmed its closest similarity to NADC30 (Figure 5). The GP5 glycoprotein of JS‐YZ/2023, belonging to Lineage 1.8, displayed identical epitope residues to its lineage prototype and only minimal divergence within the PNE epitope (Figure 5B). It also possesses four N‐glycosylation sites, modifications known to influence immune evasion and pathogenicity (Table 3).

Reverse genetics enabled the development of a recombinant virus, rGFP‐JS‐YZ/2023, which contains the full JS‐YZ/2023 genome (Figure 1) and stably expresses GFP (Figure 3A). This demonstrates that the region between ORF1b and ORF2 is insertion‐tolerant in PRRSV, validating NADC30‐like PRRSV as a flexible genetic engineering platform (Figure 3). The GFP marker allows direct visualization of viral infection dynamics, supporting precise studies of pathogenic mechanisms such as cellular changes and immune interactions. Infectious clone technology also facilitates the development of novel PRRSV vaccines [36]. By inserting or deleting specific genes within the PRRSV genome, recombinant viruses with attenuated or nontoxic properties can be generated as live vaccine candidates [37].

PCR testing excluded common porcine diarrheal viruses and Getah virus. Piglets challenged with the novel recombinant strain JS‐YZ/2023 exhibited clinical signs including diarrhea, fever, and significantly reduced average daily weight gain (Figure 6A–C). This contrasts with NADC30‐like strains, which typically cause only brief low fever and minimal weight reduction [38], and with HP‐PRRSV strains such as HuN4, which induce severe high fever, sharp declines in weight gain, and high mortality [39]. JS‐YZ/2023 also displayed distinct transmission dynamics. Unlike classical NADC30 strains, which primarily shed via nasal routes, JS‐YZ/2023 demonstrated atypical fecal–oral transmission potential, with viral loads higher in anal swabs than in respiratory samples. The associated diarrhea suggests intestinal immune evasion and mucosal damage, impairing digestion, absorption, and barrier function, thereby increasing susceptibility to secondary infections. Viremia was detectable as early as 1 dpi and rose rapidly (Figure 6H). This early viremia pattern resembles HP‐PRRSV strains but differs from the classical VR2332 strain, where viremia is typically detected at 3–5 dpi [38, 39]. The excretion pattern of JS‐YZ/2023 was unique, combining early dissemination characteristics of HP‐PRRSV with the persistent infection traits of NADC30‐like strains. Seroconversion occurred by 9 dpi, indicating rapid induction of specific antibodies that remained at high levels (Figure 6I). This differs from NADC30‐like strains, which usually seroconvert at 7–10 dpi with lower titers, HP‐PRRSV strains, which seroconvert earlier (5–7 dpi) with high titers, and NADC34‐like strains, which show delayed and weaker antibody responses [38, 39]. Following inoculation via intramuscular and nasal routes, JS‐YZ/2023 initially colonized the tonsils and then spread through lymphatic circulation to lymph nodes, resulting in high viral loads at these sites and promoting systemic persistence [40, 41]. Although the lungs are a primary target organ for PRRSV, viral loads there were lower than in the tonsils and lymph nodes [42]. This may reflect robust—though damaging—local inflammatory responses in the lungs that enhance viral clearance relative to lymphoid tissues (Figure 6J).

Normal piglet lungs appeared pink, soft, and flexible, indicating good circulation (Figure 7F). Histologically, H&E staining revealed thin, intact alveolar walls with minimal exudate or cellular infiltration (Figure 7G). By contrast, piglets challenged with JS‐YZ/2023 exhibited lungs with extensive gray–black areas and red spots; the tissue appeared hard and moist (Figure 7A). H&E staining confirmed interstitial pneumonia, characterized by thickened alveolar walls, dilated and congested blood vessels, and substantial inflammatory cell infiltration within the alveolar walls and stroma (Figure 7B). Immunohistochemistry revealed positive brown–red macrophages in lung tissue (Figure 7D–E), with staining primarily cytoplasmic and occasionally granular. Faint positive staining was also sometimes observed within vascular structures. These pathological changes caused tissue damage and dysfunction, impairing both respiratory and immune function. Normal piglet intestines were light red, soft, elastic, with thin transparent walls, a spiral disk conformation, and smooth mucosa with intact villi (Figure 8F). H&E staining showed a clear intestinal structure, loose submucosal connective tissue, and minimal inflammatory cells without significant infiltration (Figure 8G). By contrast, virus‐challenged piglets displayed thinned intestinal walls, dehydration, and loss of elasticity (Figure 8A). H&E staining revealed marked inflammatory cell infiltration, villous shortening, thickening, and fusion, impairing absorptive function. Infected intestinal cells varied in size and shape, with shrunken nuclei, and showed evidence of both mitotic activity and cell dissolution (Figure 8B). Immunohistochemistry confirmed abundant PRRSV antigen accumulation near crypt glands (Figure 8D).

PRRSV infection triggers a complex inflammatory cascade involving cytokine expression and activation of signaling pathways [43]. While serum cytokine levels reflect systemic inflammation, tissue‐specific mRNA quantification more accurately characterizes localized immune responses at viral replication sites [44]. In PRRSV pathogenesis, dysregulated cytokine responses in lymphoid organs, such as mediastinal or mesenteric lymph nodes, are closely associated with viral persistence and immunopathology [45]. Analysis of cytokine concentrations in infected piglet tissues revealed significantly higher levels in the intestine and mesenteric lymph nodes than in the lungs, liver, and spleen (Figure 9). This indicates that PRRSV infects and replicates within intestinal tissues and associated lymph nodes, driving a more intense localized inflammatory response [46]. These sites contain abundant immune cells such as macrophages and lymphocytes, which produce inflammatory factors. Elevated IFN‐α (Figure 9E) and IFN‐γ (Figure 9F) levels specifically in mesenteric lymph nodes point to heightened antiviral and cell‐mediated immune activity within this tissue. By contrast, relatively lower cytokine levels in lungs, liver, and spleen suggest either weaker immune responses or lower viral burden in these organs. PRRSV infection is also known to activate inflammatory signaling pathways mediated by Toll‐like receptors—particularly TLR4—and RIG‐I‐like receptors [47]. These pathways may be more active within immune cells of the gut and mesenteric lymph nodes, contributing to the elevated cytokine levels observed in these tissues [48]. Overall, the immune response to PRRSV infection varies significantly across tissues, with the intestine and mesenteric lymph nodes mounting the strongest responses.

The PRRSV strain JS‐YZ/2023 demonstrates a “clinically mild but pathologically persistent” dual pathogenicity. Its virulence is markedly attenuated compared with typical HP‐PRRSV strains such as HuN4, which cause 40%–100% mortality, and the highly pathogenic recombinant strain GD‐7, reported to cause 33.3% mortality in a 2023 study [49]. This attenuation likely results from NADC30‐like recombination within the NSP1–2 region. Such recombination may balance the strong replication capacity of the HP‐PRRSV backbone with NADC30‐derived immunomodulatory proteins, thereby preventing excessive inflammation. A notable feature of JS‐YZ/2023 is its distinct intestinal tropism, evidenced by high viral loads in anal swabs that correlate with infection of intestinal crypts. While rare in HP‐PRRSV, similar intestinal tropism has been described in PRRSV‐1 strains [50]. Altered GP5 receptor‐binding specificity may underlie this phenotype, although the precise mechanism remains to be clarified.

## 5. Conclusions

This study isolated and characterized JS‐YZ/2023, a novel recombinant PRRSV. Genetic analysis showed that JS‐YZ/2023 carries a high‐pathogenicity PRRSV backbone that has recombined with NADC30‐like strains at the Nsp1–2 and ORF4–6 genomic regions. Using reverse genetics, we successfully generated rGFP‐JS‐YZ/2023, which stably expressed GFP. Growth kinetics analysis demonstrated that rGFP‐JS‐YZ/2023 replicated comparably to both JS‐YZ/2023 and the rescued strain rJS‐YZ/2023. Pathogenicity assessment in 1‐month‐old weaned piglets confirmed JS‐YZ/2023 as moderately virulent. Infected piglets developed interstitial pneumonia along with diarrhea, and strong PRRSV signals were detected within the intestinal mucosa. Overall, this work advances understanding of NADC30‐like PRRSV evolution and molecular epidemiology in China, providing critical insights for the development of effective national control strategies against PRRSV.

## Ethics Statement

Animal experiments were approved by the Shanghai Veterinary Research Institute IACUC (SV‐20241101‐03) and conducted in accordance with China’s Humane Treatment of Laboratory Animals guidelines (MOST Policy 2006398). All procedures complied with national ethical standards.

## Conflicts of Interest

The authors declare no conflicts of interest.

## Author Contributions

**Yafang Lin and Yan Ouyang**: sequence analysis, animal experiments, manuscript drafting. **Jiayang Zheng and Changguang Xiao:** JS‐YZ/2023 sequencing, variation analysis. **Yang Yang**, **Qianming Zhao, and Yan Zhang:** qRT‐PCR, ELISA, data analysis. **Zongjie Li, Ke Liu, Donghua Shao, Beibei Li, and Yafeng Qiu:** manuscript revision. **Zhiyong Ma and Jianchao Wei:** experimental design, manuscript revision. All authors: final editing and approval. Yafang Lin and Yan Ouyang contributed equally to this work and are co‐first authors.

## Funding

This work was supported by the National Key Research and Development Program of China (Grant 2022YFD1800801, awarded to Jianchao Wei) and the Central Public‐interest Scientific Institution Basal Research Fund (Grant CAAS‐ZDRW202409, awarded to Jianchao Wei).

## Data Availability

Data are available from the corresponding author upon reasonable request.
